# Mapping the longitudinal trajectories of postural control and functional activity after stroke using clinical and sensor-based assessments: protocol of the FOSTER prospective cohort study

**DOI:** 10.1136/bmjopen-2026-121574

**Published:** 2026-08-17

**Authors:** Charlotte Moeyersons, Elissa Embrechts, Britta Hanssen, Mahyar Firouzi, Sylvie De Raedt, Lubos Omelina, Bernard Dan, Sandra Ortega-Martorell, Bart Jansen, Marc Degelaen, Eva Swinnen, TARGET consortium

**Affiliations:** 1 Rehabilitation Research Group (RERE), Department of Physiotherapy, Human Physiology and Anatomy, Vrije Universiteit Brussel, Brussels, Belgium; 2 MOVANT Research Group, Department of Rehabilitation Sciences and Physical Therapy, Faculty of Medicine and Health Sciences, University of Antwerp, Antwerp, Belgium; 3 Inkendaal Rehabilitation Hospital, Sint-Pieters-Leeuw, Belgium; 4 Department of Neurology, Universitair Ziekenhuis Brussel (UZ Brussel), NEUR Research Group, Vrije Universiteit Brussel (VUB), Brussels, Belgium; 5 Department of Electronics and Informatics (ETRO), Engineering Sciences, Vrije Universiteit Brussel, Brussels, Belgium; 6 Faculty of Psychology, Educational Sciences and Speech and Language therapy (CP122), Universite Libre de Bruxelles, Brussels, Belgium; 7 Artificial Intelligence and Digital Technologies Research Institute, Liverpool John Moores University, Liverpool, UK; 8 IMEC, Leuven, Belgium

**Keywords:** Stroke, Longitudinal studies, REHABILITATION MEDICINE

## Abstract

**Abstract:**

**Introduction:**

Stroke is a leading cause of long-term disability, yet recovery trajectories are highly heterogeneous and difficult to predict at the individual level. Standardised clinical assessments primarily capture task accomplishment under controlled conditions but may overlook changes in movement quality, including the distinction between compensatory strategies and behavioural restitution. Additionally, they provide limited insight into the postural control processes that underlie functional task performance. Recent consensus initiatives emphasised the need for sensitive, functionally relevant measures of balance and mobility after stroke. Integrating routine clinical assessments with a sensor-based approach during functional tasks offers new opportunities to characterise recovery more precisely and to map longitudinal trajectories of poststroke change. Within the European Union-funded TARGET project, the FOSTER study aims to generate multimodal data to support data-driven, individualised prognostic models, so-called virtual twins, for personalised rehabilitation.

**Methods and analysis:**

FOSTER is a prospective, single-centre observational cohort study at Rehabilitation Hospital Inkendaal (Belgium). We aim to include 100 adults within 8 weeks poststroke, assessed at three to five time points, including an entry assessment after admission and follow-up assessments within predefined windows at approximately 5, 8, 12 and 24 weeks poststroke. The protocol combines synchronised sensor-based measurements during functional tasks (seated reaching, standing reaching and gait initiation) obtained during an exergame, with standardised clinical tests and patient-reported outcome measures (PROMs). Sensors include surface electromyography, inertial measurement units, force plates and markerless motion capture. Sensor-based measurements target movement quality, and anticipatory and reactive postural control during self-initiated functional tasks. Derived outcomes will capture kinetic, kinematic and neuromuscular features of movement strategies, including temporal and asymmetry characteristics. In addition, in-task performance metrics, real-world activity monitoring through accelerometry, standardised clinical assessments and PROMs will be collected to provide a comprehensive, multimodal characterisation of recovery. Linear mixed-effects models will characterise longitudinal trajectories and relationships between clinical and sensor-derived measures.

**Ethics and dissemination:**

The FOSTER study was approved by the Ethics Committee of Rehabilitation Hospital Inkendaal (B.U.N. 2024-TEE-002). Participants will be included after providing informed consent. Data are pseudonymised and stored on a General Data Protection Regulation-compliant platform. Findings will be disseminated through peer-reviewed publications, conferences and within the TARGET consortium.

**Trial registration number:**

NCT06806761.

**STRENGTHS AND LIMITATIONS OF THIS STUDY**

- Prospective, longitudinal protocol with three to five predefined time points in the first 6 months poststroke.
- Task-specific, game-based assessments designed to elicit natural movement while maintaining standardisation across sessions.
- Integration of multiple sensor modalities (surface electromyography, inertial measurement units, force plates, markerless motion capture) enables objective quantification of postural control and movement quality.
- Single-centre, observational design may limit generalisability and precludes causal inference.
- Repeated multisensor assessments may increase participant burden and missing data, particularly among more impaired individuals.

## Introduction

 Stroke remains one of the leading causes of long-term disability worldwide, with substantial societal and individual burdens. In Europe alone, over one million people experience a stroke annually. Stroke is typically accompanied by interacting deficits like muscle weakness and impaired postural control, often resulting in persistent limitations in mobility and reduced functional independence.^1–3^ Recovery of mobility largely depends on both the restoration of lower extremity function and the ability to regain adequate postural control.^4 5^ Despite well-established evidence-based rehabilitation guidelines, recovery trajectories remain highly heterogeneous and difficult to predict at the individual level, posing a major clinical challenge.^6 7^ Recent advances in artificial intelligence have been proposed as a promising avenue to address this challenge by integrating heterogeneous clinical and physiological data to support personalised prediction and management strategies across the atrial fibrillation (AF-)stroke continuum.^8^

Standardised clinical assessments, such as the Fugl-Meyer assessment (FMA), Berg Balance Scale (BBS) or Functional Independence Measure (FIM), play a vital role in monitoring rehabilitation progress and guiding clinical decision-making.^4 9^ These instruments provide structured evaluations of motor impairment, balance and functional independence and are widely implemented in both research and clinical practice.^4^ Most existing prediction models for motor recovery, including the Predict Recovery Potential 2 (PREP2) algorithm for the upper limb^10^ and the TWIST algorithms for ambulation,^11^ are largely dependent on baseline demographic characteristics and clinical assessment scores, making their prognostic value dependent on the scope and sensitivity of these measures. While valuable for early stratification and outcome monitoring, clinical assessments primarily evaluate task accomplishment under controlled conditions.^12–19^

Beyond task accomplishment, a limitation of standardised clinical assessments is that they provide limited insight into movement quality, as they are unable to distinguish behavioural restitution, that is, re-acquisition of prestroke movement patterns, from compensatory strategies, that is, substitution for, or circumvention of, impaired functions.^20 21^ Additionally, they provide limited insight into the mechanisms underlying postural control, which involves multisensory integration and the interaction between anticipatory (feedforward) and reactive (feedback) processes.^22–24^ Anticipatory (feedforward) postural control prepares the body for self-generated disturbances, whereas reactive (feedback) control restores stability following movement execution.^25–27^ Together, this highlights the need for complementary assessment approaches with greater sensitivity to underlying movement quality, as emphasised by recent international consensus initiatives on poststroke outcome measurement.^4 9 28–30^

Sensor technologies, especially wearable ones, offer promising opportunities to address these limitations by enabling objective, fine-grained and even continuous measurement of movement and postural control during functional tasks.^31–34^ Depending on the modality, sensors such as force plates, surface electromyography (sEMG), and inertial measurement units (IMUs) provide complementary information on kinetics (eg, asymmetric weight distribution), muscle activation patterns (eg, delayed muscle activation) and kinematics (eg, excessive trunk displacement). By quantifying these dimensions, sensor-based assessments enable more detailed characterisation of underlying movement quality and compensatory strategies. Importantly, especially wearable sensors facilitate measurement within functionally relevant contexts while maintaining standardisation and reproducibility, thereby helping bridge the gap between standardised clinical testing and functionally representative movement assessment.^35 36^

Although previous studies have demonstrated the potential of (wearable) sensor technologies to capture impairments in underlying movement quality and postural control after stroke,^34 37–40^ most investigations have been cross-sectional or confined to controlled laboratory settings.^41–43^ As a result, they provide only a glance at recovery and do not capture the temporal dynamics that characterise functional improvement. This limitation is particularly relevant given that poststroke recovery follows a non-linear trajectory, typically characterised by early gains followed by periods of plateau and consolidation.^44 45^ Capturing such dynamic changes requires longitudinal assessments that extend into clinically relevant phases of recovery.

To address this, the FOSTER (‘Follow-up study of stroke patients using sensors for the evaluation of functional ability and compensatory movements’) study was initiated within the EU-funded TARGET project (‘Health virtual twins for the personalised management of stroke related to atrial fibrillation’; grant agreement: 101136244).^46 47^ TARGET aims to improve outcomes after AF-related stroke through the development of individualised ‘health virtual twin’ models, that is, AI-driven simulations designed to support personalised clinical decision-making across the care pathway, from prevention and acute management to rehabilitation and long-term follow-up.^46 47^ Complementing this objective, FOSTER broadens the scope to poststroke recovery across all aetiologies, with prospective follow-up up to 6 months poststroke.

The overarching aim of the FOSTER clinical study is to collect longitudinal data on postural control and functional activity during the subacute phase after stroke. A multimodal assessment protocol will be applied that combines sensor-based assessments during functional activities, allowing the distinction between behavioural restitution and compensatory mechanisms, with standard clinical outcome measures. The primary objective is to characterise longitudinal change in sensor-derived postural control and movement quality. This primary outcome construct is operationalised across four equally ranked outcome domains: postural control, weight distribution, movement coordination and neuromuscular activity. Secondary objectives are to characterise longitudinal change in task-performance measures, daily activity, clinical outcomes and patient-reported outcome measures (PROMs), and to examine associations between sensor-derived metrics of movement quality and clinical outcome measures.

## Methods and analysis

### Project status

Patient recruitment and assessments started in January 2025 at Rehabilitation Hospital Inkendaal. By July 2026, halfway through the planned 36-month data collection period, 56 participants had been included and both recruitment and follow-up assessments were ongoing.

### Study design and setting

FOSTER is a prospective, single-centre observational cohort study conducted at the Rehabilitation Hospital Inkendaal, a specialised rehabilitation hospital located in Vlezenbeek, Belgium. The rehabilitation hospital includes two subacute neurological rehabilitation units with a total capacity of 86 beds, providing a structured setting for standardised interdisciplinary follow-up after stroke. Rehabilitation is highly individualised and delivered according to standardised care pathways, integrating physiotherapy, occupational therapy, speech and language therapy and medical follow-up tailored to each patient’s needs.

Patients are referred to Rehabilitation Hospital Inkendaal for intensive rehabilitation following acute care in a primary hospital. The study was designed to fit within the existing care trajectory for stroke rehabilitation, ensuring clinical feasibility while enabling structured data acquisition for scientific analysis. Participants are enrolled during their inpatient stay and followed up longitudinally throughout the rehabilitation process.

The study was developed in accordance with the STROBE (Strengthening the Reporting of Observational Studies in Epidemiology) guidelines for observational studies^48^ and ethical approval was granted by the local ethics committee at Rehabilitation Hospital Inkendaal (B.U.N. 2024-TEE-002).

### Patient and public involvement

Patients and members of the public were not involved in the design of the FOSTER study. However, within the wider TARGET project, a dedicated stakeholder work package has been established to ensure that the development of AI-based clinical decision-support tools reflects the needs and perspectives of end users. This programme includes co-creation activities with clinicians, patients and other stakeholders through advisory boards, focus groups, and iterative feedback processes, which inform the design and implementation of the TARGET digital tools.^46 47^

### Participants

#### Eligibility criteria

Participants are adults admitted to Rehabilitation Hospital Inkendaal for inpatient stroke rehabilitation. Recruitment is embedded in the hospital intake pathway; the multidisciplinary team flags potentially eligible patients to the research team who subsequently provides verbal and written study information and answers questions (with family present if desired). Written informed consent is obtained by the research team before any study-specific assessments.

**Figure 1:**
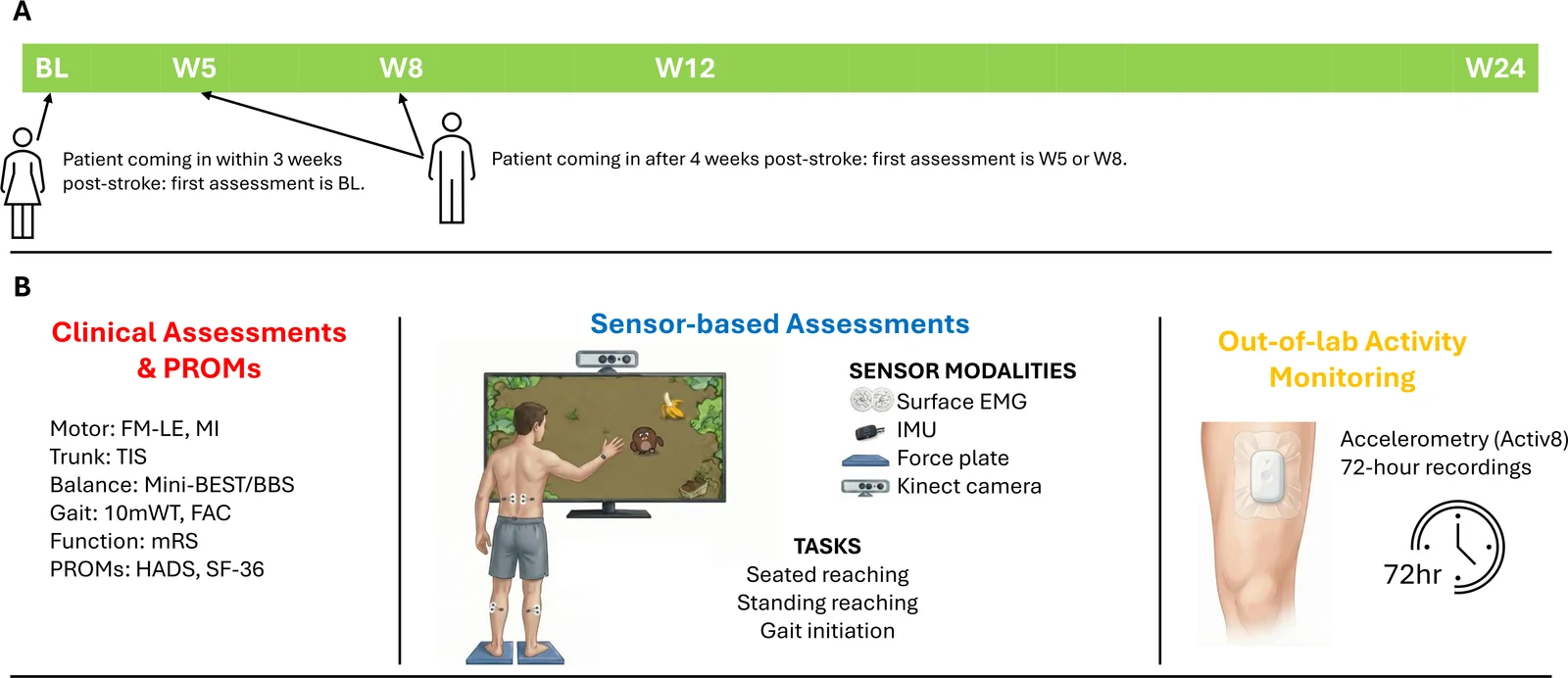
Overview of the FOSTER multimodal longitudinal assessment protocol (**A**) Assessment timeline; 3–5 assessments per participant (depending on inclusion timing) (**B**) Multimodal assessment approach; the central panel includes the in-game visual interface where the on-screen avatar, controlled by the participant’s non-paretic hand, reaches towards the target (banana). 10mWT, 10 m Walk Test; BBS, Berg Balance Scale; BL, baseline; EMG, electromyography; FAC, Functional Ambulation Category; FMA-LE, Fugl-Meyer Assessment–Lower Extremity; HADS, Hospital Anxiety and Depression Scale; IMU, inertial measurement unit; MI, Motricity Index; Mini-BEST, Mini-Balance Evaluation Systems Test; mRS, modified Rankin Scale; PROMs, Patient-Reported Outcome Measures; SF-36, 36-Item Short Form Health Survey; TIS, Trunk Impairment Scale; W, Week.

To be eligible, adults (≥18 years old) must have experienced a supratentorial stroke (ischaemic or haemorrhagic) and be within the first 8 weeks poststroke onset at the time of inclusion. This time frame ensures that participants can complete at least three poststroke assessments (at 8, 12 and 24 weeks), in line with the study’s longitudinal design (see figure 1). The FOSTER cohort is not restricted to patients with AF-related stroke; patients are eligible irrespective of stroke aetiology, provided they meet the other eligibility criteria. Participants must also be capable of understanding and performing the study procedures that are feasible at a given assessment time point, following the advice of the treating therapist(s) and/or physician(s).

Patients are not eligible if they present with neurological or orthopaedic comorbidities unrelated to stroke that significantly impair motor function or if they have severe medical conditions that could compromise safety during the assessments. Additional exclusion criteria include major deficits in communication, memory or understanding, as well as insufficient comprehension of Dutch, French, German or English. Independent walking, standing balance or the ability to perform the exergame tasks are not required for inclusion.

### Assessment protocol

Each participant undergoes an entry visit (first study assessment) as soon as feasible after admission and informed consent. Follow-up assessments are scheduled within predefined poststroke windows at 5, 8, 12 and 24 weeks after stroke onset. As participants are eligible for inclusion within the first 8 weeks poststroke, they will complete between three and five assessments depending on their admission timing. Participants admitted later in the recovery trajectory (eg, around week 7–8 poststroke) will be scheduled at the first possible window and will subsequently be followed at the remaining scheduled windows (eg, weeks 8, 12 and 24) (figure 1).

All assessments are conducted by trained researchers, under standardised conditions in the hospital’s motion analysis lab (see further). Sessions are integrated into the rehabilitation schedule to minimise interference with therapy and last up to 120 min, including rest breaks as needed. When participants are discharged before a scheduled time point, outpatient follow-up is arranged whenever feasible.

The protocol consists of two components: (1) a series of structured, sensor-based recordings during functional activities obtained through an exergame and (2) a standardised set of clinical tests and PROMs, conducted without sensors. The clinical assessments and PROMs are completed whenever feasible, whereas the sensor-based exergame assessments are only performed when the participant is judged able to complete the respective task safely under supervision.

The sensor-based assessments are performed through an interactive game environment. The game interface (figure 1B) visualises an avatar, controlled by the participant’s non-paretic hand or a leg, who can move toward a target object. This game uses Kinect V2 markerless motion capture, placed in front of the participant, to track full-body movements in the frontal plane. Visual targets are presented on a screen to elicit controlled reaching or stepping movements. These tasks are designed to safely challenge postural control through controlled destabilisation and to elicit natural, functionally relevant movement responses.

The game embeds three core functional tasks: (1) Seated reaching: sideways reaching from a standardised seated position toward a virtual target; (2) Standing reaching: the same reaching task performed while standing; and (3) Gait initiation: initiating forward stepping in response to a visual target. Figure 1 illustrates the standing reaching condition. Reaching is always performed with the non-paretic arm, both to the paretic and non-paretic side. Stepping is performed with both the paretic and non-paretic leg as stepping leg. A minimum of 5 targets per side is collected for each functional task.

The game consists of two different levels, presented in the same order in every session. In the first level, the participant knows the location of the target in advance and waits with initiating the requested movement until a timer clock has counted down. In the second level, the target location is unknown until it appears on the screen, which is also the sign to initiate the movement.

Rehabilitation hospital Inkendaal is equipped with a motion analysis laboratory (±40 m²) including AMTI force platforms (Advanced Mechanical Technology, Watertown, Massachusetts, USA), wireless Mini Wave Infinity modules for sEMG and IMU recording (Cometa s.r.l., Bareggio (MI), Italy), four Bonita 720C video cameras (Vicon Motion Systems, Oxford, UK) and exergame-related markerless motion capture using Kinect V2 (Microsoft Corporation, Redmond, WA, USA). Multimodal acquisition is coordinated through Vicon Nexus (V.2.16; Vicon Motion Systems, Oxford, UK), enabling time-synchronised recordings across all data streams.

The full experimental laboratory setup, including all sensor modalities, is illustrated in figure 1B. sEMG is used to record bilateral muscle activity from key postural and lower limb muscles, including the tibialis anterior, lateral gastrocnemius, soleus, gluteus medius, biceps femoris and erector spinae. Electrode placement follows SENIAM guidelines in combination with palpation.^49^ In seated reaching, only the erector spinae will be evaluated. IMUs are placed on the sternum, pelvis and non-paretic wrist. Additionally, during standing reaching and gait initiation, force plate data are collected with each foot placed on a separate force plate.

Tasks are executed according to a standardised protocol in terms of verbal instruction, trial sequence and rest intervals. The same setup and procedures are applied across all time points to ensure consistent longitudinal data.

The clinical assessments and PROMs, conducted without sensor instrumentation, include standardised tests to assess lower extremity function, trunk control, balance, gait capacity, functional independence, mental health and quality of life. In addition, daily physical activity will be monitored using an accelerometer (Activ8 Professional; 2M Engineering; The Netherlands), worn on the anterior upper leg for 72 consecutive hours at each assessment time point. The device may be worn on either the affected or unaffected side, as physical activity classification is not expected to differ meaningfully between sides.

### Outcome measures

Outcome measures are collected at the entry visit and each applicable poststroke time point, according to the patient’s ability. Depending on functional ability, not all components may be feasible at early assessments. Participants who are not yet able to perform the exergame tasks will complete the feasible clinical assessments and PROMs only, and sensor-based exergame assessments will be added at later time points when they can be performed safely. For example, seated reaching may become feasible before standing reaching and gait initiation as motor ability improves. An overview of all outcome measures, including data sources, is provided in table 1.

**Table 1:** Outcome measures, instruments and data sources

Outcome category	Outcome/variable	Data source
Patient characteristics		
	Sociodemographic profile (age, sex, occupation, ethnicity, education)	Medical record
	Stroke descriptors and relevant comorbidities (lesion side and site, type, aetiology, AF status/subtype)	Medical record
	Acute stroke severity	NIHSS
	Global cognition	MoCA
Sensor-based outcomes		
Exergame assessments	Postural control* (eg, postural sway)	IMUs; force plates
	Weight distribution*	Force plates
	Neuromuscular activity*	sEMG
	Movement coordination*	IMUs; Kinect; force plates
	Task accuracy (reach/step)	Kinect
	Upper-limb acceleration	IMUs
	Reaction and movement time	Kinect
Out-of-lab monitoring	Daily activity profile	Activ8 sensor
Clinical and patient-reported outcomes		
Clinical assessments	Lower-limb motor impairment	FM-LE (0–34); MI (0–100)
	Dynamic sitting balance	TIS (0–6)
	Standing balance	Mini-BEST (0–28) or BBS (0–56)
	Walking speed	10mWT (m/s)
	Ambulation category	FAC (0–5)
	Global disability	mRS (0–5)
PROMs	Anxiety and depression	HADS (0–21 subscores)
	Quality of life (health-related)	SF-36 (domain scores 0–100)

* Primary outcome domain. The four primary outcome domains are equally ranked, with no further hierarchy. All other outcome measures are secondary.

AF, atrial fibrillation; BBS, Berg Balance Scale; FAC, Functional Ambulation Categories; FM-LE, Fugl–Meyer Assessment-Lower Extremity; HADS, Hospital Anxiety and Depression Scale; IMUs, inertial measurement units; MI, Motricity Index; Mini-BESTest, Mini-Balance Evaluation Systems Test; MoCA, Montreal Cognitive Assessment; mRS, modified Rankin Scale; 10mWT, 10 m Walk Test; NIHSS, National Institutes of Health Stroke Scale; PROMs, patient-reported outcome measures; sEMG, surface electromyography; SF-36, 36 Item Short Form Health Survey; TIS, Trunk Impairment Scale.

#### Sociodemographic and clinical characteristics

At the entry visit, data are collected to describe the study population and support exploratory analyses. Sociodemographic and clinical information are extracted from medical records, including age, biological sex, occupation, ethnicity, educational level, lesion side and site, stroke type and aetiology, relevant comorbidities, and where documented, AF status and subtype and the National Institutes of Health Stroke Scale score from the acute hospital. Cognitive function at admission is screened using the Montreal Cognitive Assessment, a validated tool covering multiple cognitive domains.^50 51^

#### Sensor-based outcome measures

Sensor-based outcome measures are derived from the recordings obtained during seated reaching, standing reaching and gait initiation. Across the two levels of the game, these outcome measures capture anticipatory and reactive postural control, assessed both prior to and during the self-initiated functional tasks. Attention is given to both the timing and amplitude of postural responses and neuromuscular activity. Weight distribution and related asymmetry metrics will be used to quantify loading differences between the paretic and non-paretic sides. Movement coordination will be characterised by the relative timing between arm, trunk and pelvis motion during reaching and, during standing tasks, between postural weight shift and the reaching or stepping movement. Together, these outcomes provide quantitative markers of movement strategies that may enable examination of behavioural restitution and compensatory adaptations over time.^28 52^

Examples of modality-specific outcome measures include, among others, parameters derived from force plates such as centre-of-pressure trajectories, ground reaction forces and weight distribution. IMUs capture segmental accelerations and orientation-based estimates of postural sway, while sEMG signals provide muscle activation onset, amplitude and co-activation patterns. Kinect recordings capture segmental kinematics in the frontal plane, reaction and movement time and task accuracy. Together, this integrated setup enables the extraction of synchronised kinetic, kinematic and neuromuscular outcomes during each functional task. Out-of-lab monitoring is performed using a wearable activity sensor (Activ8), which enables continuous assessment of free-living movement behaviour outside the controlled laboratory environment. Daily activity, quantifying the time spent sitting, standing and walking, will be assessed during a 72-hour period.

#### Clinical and PROMs

The clinical test battery includes standardised instruments to assess lower extremity function and motor impairment, including balance, gait, strength, functional independence and psychosocial well-being. Lower limb motor performance is evaluated using the FMA–Lower Extremity and the Motricity Index. Both instruments demonstrate excellent reliability in stroke populations.^53–56^ Dynamic sitting balance is assessed with the dynamic subtest of the Trunk Impairment Scale, a validated and reliable tool recommended for stroke rehabilitation.^4 57^ Standing balance is evaluated using either the Mini-Balance Evaluation Systems Test and/or the BBS*,* depending on ambulatory capacity. Both scales have strong psychometric properties.^4 58 59^

Walking performance and independence are assessed using the 10 m Walk Test and the Functional Ambulation Category, which are reliable, valid and widely used in stroke rehabilitation.^60–63^ Global functional status is documented using the Modified Rankin Scale, widely used in clinical trials for its simplicity and reliability in measuring poststroke disability.^64^ Mental health and health-related quality of life are assessed with the Hospital Anxiety and Depression Scale and the 36-item Short Form Survey, both of which are validated instruments with good reliability in neurological populations.^65 66^

### Data analyses

#### Sample size

Assessments are expected to take place over a 36-month period, and the study aims to enrol 100 participants. This target was defined pragmatically based on the average admission flow at the rehabilitation site, where approximately seven patients per month are expected to be screened, of whom an average of three are expected to meet the eligibility criteria and be included. The planned sample is considered appropriate for the observational design of the study and its objective of describing longitudinal recovery trajectories across repeated assessments in a real-world rehabilitation cohort.

#### Statistical analysis

For the primary objective, longitudinal change in outcomes representing the four primary sensor-based domains (postural control, weight distribution, movement coordination and neuromuscular activity) will be analysed using linear mixed-effects models. Secondary longitudinal analyses will apply the same modelling framework to task-performance measures, daily activity, clinical outcomes and PROMs. Baseline characteristics will be summarised descriptively. Feasibility and data availability will be reported per assessment timepoint, documenting (1) the proportion of participants completing each assessment, (2) the number of valid trials per condition for sensor-based tasks and (3) reasons for missing data at each timepoint.

To investigate longitudinal change, each outcome will be modelled as a function of Time (categorical: baseline, week 5, week 8, week 12 and week 24). Models will include subject-specific random intercepts to account for baseline differences between individuals. Where supported by convergence and reliable variance estimation, random slopes for Time will be added to capture heterogeneity in individual recovery trajectories.^67 68^ Estimated marginal means will be used to describe group-level recovery trajectories, and post hoc contrasts will quantify differences across the full study period and between assessment intervals. Within each outcome domain, multiplicity will be controlled using Holm-Bonferroni correction.

Individual predicted trajectories will be visualised by combining individual intercept and slope estimates with the fixed effects. These individual trajectories will be plotted alongside group-level trajectories to illustrate both average trends and the distribution of recovery profiles across participants. Standard diagnostic checks will be performed; if assumptions are violated, transformations or alternative model specifications will be considered.

To examine associations between sensor-derived metrics of movement quality and clinical outcome measures, a multivariate mixed-effects modelling approach will be used. First, separate linear mixed-effects models will be fitted for each individual outcome, using the same model structure described above for the longitudinal analyses. These models provide subject-specific random intercepts and, when supported by convergence, random slopes for Time, representing each participant’s baseline level and rate of change.

Next, bivariate (joint) mixed-effects models will be constructed to quantify the association between selected pairs of outcomes, that is, one sensor-derived metric and one clinical measure. Pairings will be defined based on theoretical considerations and clinical relevance. These models estimate the covariance between the random effects of the two outcomes, from which we will estimate (1) the correlation between random intercepts, reflecting the association between participants’ baseline sensor-based movement quality and their baseline clinical performance and (2) the correlation between random slopes, reflecting whether improvements in sensor-derived outcomes correspond to improvements on clinical measures over time.

Correlations will be interpreted descriptively using conventional thresholds (eg, negligible, low, moderate, high, very high). Because this aim is exploratory, α=0.05 will be used without correction for multiple comparisons. Associations will be visualised using scatterplots of individual random effects and bivariate trajectory plots.

## Ethics and dissemination

The study is conducted in accordance with the Declaration of Helsinki and the General Data Protection Regulation (EU 2016/679). Ethical approval was granted by the Ethics Committee of Rehabilitation Hospital Inkendaal (B.U.N. 2024-TEE-002). The study is registered at ClinicalTrials.gov (NCT06806761). Potential participants receive oral and written information about the study’s objectives and procedures before providing written informed consent; where decision-making capacity is lacking due to language or cognitive impairment, consent will be obtained from a legally authorised representative in line with Belgian legislation. Participants who later regain capacity will be re-consented (within the ≤8-week window).

Assessments involve low-risk functional tasks under direct supervision by a licensed physiotherapist; participants may pause or stop at any time. Predefined stopping criteria apply (eg, fatigue/instability, near-fall, dizziness, chest pain or clinician concern). Adverse events are recorded; serious events are reported per institutional policy.

Results will be presented at national and international scientific meetings and submitted to peer-reviewed journals; institutional websites and media offices may also be used. Pseudonymised data and metadata will be deposited in a secure institutional repository and on the TARGET data platform after study completion under controlled access and curated according to the study’s data management plan; external requests will be considered via the corresponding author under a data-use agreement.

## Discussion

The FOSTER study is expected to provide longitudinal, multimodal data on postural control, movement quality, functional activity and PROMs during the first 6 months after stroke. By combining standardised clinical assessments with sensor-based measurements during functional tasks, the study may provide insight into aspects of recovery that are not fully captured by clinical scales alone, including changes in movement strategies and postural control during task performance. These data may help identify candidate sensor-derived markers of poststroke recovery and contribute rehabilitation-related data to the wider TARGET project.

Several practical challenges and limitations are anticipated. The single-centre, observational design may limit generalisability and preclude causal inference. Repeated multimodal assessments may also increase participant burden, particularly in people with fatigue, cognitive difficulties or more severe motor impairment. In addition, not all participants will be able to complete all sensor-based exergame tasks at each time point, especially early after stroke. Follow-up after discharge, particularly at 24 weeks, may be challenging because participants may need to return to the hospital.

To reduce these challenges, assessments are scheduled flexibly, rest breaks are provided when needed and sensor-based tasks are only performed when they are safe and feasible. After discharge, participants are asked whether they are willing and able to continue follow-up and participation remains entirely voluntary. When participants continue outpatient rehabilitation at the hospital, follow-up assessments are aligned with their scheduled appointments whenever possible. Travel costs are not reimbursed, which may contribute to loss to follow-up after discharge.

By contributing longitudinal, multimodal rehabilitation data across stroke aetiologies, FOSTER will complement TARGET’s broader development of health virtual twin models for AF-related stroke. Although the FOSTER cohort is not restricted to AF-related stroke, these data may help inform future data-driven models of poststroke recovery and personalised rehabilitation.

## Supplementary material

10.1136/bmjopen-2026-121574 online supplemental file 1
