# Supplementary material for: Mapping the longitudinal trajectories of postural control and functional activity after stroke using clinical and sensor-based assessments: protocol of the FOSTER prospective cohort study
Source: BMJ Open. 2026 Aug 17;16(8):e121574. doi: 10.1136/bmjopen-2026-121574 (PMC13504868; doi:10.1136/bmjopen-2026-121574)
Supplement: Supplementary file 1 [file bmjopen-2026-121574.reviewer_comments.pdf]

PEER REVIEW HISTORY

BMJ Open publishes all reviews undertaken for accepted manuscripts. Reviewers are asked to complete a checklist review form and are provided with free text boxes to elaborate on their assessment. These free text comments are reproduced below.

ARTICLE DETAILS

Title (Provisional)

Mapping the longitudinal trajectories of postural control and functional activity after stroke using clinical and sensor-based assessments: protocol of the FOSTER prospective cohort study

Authors

Moeyersons, Charlotte; Embrechts, Elissa; Hanssen, Britta; Firouzi, Mahyar; De Raedt, Sylvie; Omelina, Lubos; Dan, Bernard; Ortega-Martorell, Sandra; Jansen, Bart; Degelaen, Marc; Swinnen, Eva; Consortium, TARGET

VERSION 1 - REVIEW

**Reviewer**	**1**
**Name**	**Brunner, Iris**
**Affiliation**	**Aarhus University**
**Date**	**13-Jun-2026**
**COI**	**None**

This protocol describes the FOSTER study, a prospective observational study on patients with stroke due to atrial fibrillation.

Using both clinical and sensory assessment over an extended period of time, the study aims to identify trajectories of recovery for lower limb, postural control and daily functioning.

The protocol is comprehensive and intelligible.

However, there are some points that could be clarified and/or elaborated

1. Are the patients to be included AF patients? This is not mentioned in the inclusion criteria.
2. Moreover, the authors do not mention gait function. Isn’t it a prerequisite for the exergame to have some gait function or balance? Please include these criteria in the eligibility criteria.

3. It is not clear what the primary outcome is. Could you specify, is there a hierarchy?

4. I would suggest completing the discussion with some sentences about the expected outcomes and what they could be good for. Moreover, what challenges are to be expected,

e.g. it must be a challenge to ask patients to come to the hospital again after 6 months. Is travel reimbursed?

VERSION 1 - AUTHOR RESPONSE

REVIEWER 1			
1	This protocol describes the FOSTER study, a prospective observational study on patients with stroke due to atrial fibrillation.<br><br>Using both clinical and sensory assessment over an extended period of time, the study aims to identify trajectories of recovery for lower limb, postural control and daily functioning.<br><br>The protocol is comprehensive and intelligible.<br><br>However, there are some points that could be clarified and/or elaborated:	We thank the reviewer for their positive and constructive assessment of our protocol.	
2	**1. Are the patients to be included AF patients?**<br><br>This is not mentioned in the inclusion criteria.	Thank you for raising this point. Although the FOSTER study is embedded within the TARGET project, which focuses on personalised management of AF-related stroke, the FOSTER cohort itself is not restricted to patients with AF-related stroke. Patients with supratentorial ischaemic or haemorrhagic stroke are eligible irrespective of stroke aetiology, provided they meet the other eligibility criteria. Stroke aetiology and relevant comorbidities, including atrial fibrillation status and subtype where available, are recorded as part of the baseline clinical descriptors.	**The eligibility criteria and patient characteristics sections have been revised** to clarify that AF-related stroke is not an inclusion criterion for FOSTER, and that stroke aetiology, relevant comorbidities and atrial fibrillation status/subtype are recorded as baseline clinical descriptors.
3	**2. Moreover, the authors do not mention gait function. Isn't it a prerequisite for the exergame to have some gait function or balance?**<br><br>Please include these criteria in the eligibility criteria.	Thank you for this important clarification. Independent gait function, standing balance, or the ability to perform the exergame tasks are not prerequisites for inclusion.<br><br>Because the FOSTER study follows patients longitudinally during recovery, participants may be included even when they are not yet able to perform the sensor-based exergame assessments. In such cases, the feasible clinical assessments and PROMs are completed, and the exergame tasks are introduced at later time points	**The eligibility criteria, assessment protocol and outcome measures sections have been revised** to clarify that independent gait function, standing balance, or the ability to perform the exergame tasks are not required for inclusion. The manuscript now specifies that sensor-based exergame assessments are administered according to functional ability and safety at each time point, while feasible clinical assessments and PROMs are completed when the exergame tasks cannot yet be performed.

		when the participant is judged able to perform them safely under supervision. The sensor-based exergame tasks are therefore only attempted when considered safe and feasible. We have clarified this in the eligibility criteria, assessment protocol and outcome measures sections.	
4	**3. It is not clear what the primary outcome is.**<br><br>Could you specify, is there a hierarchy?	Thank you for this important comment. We agree that the outcome hierarchy was not sufficiently explicit in the original manuscript. No single primary outcome measure was designated. Instead, the primary outcome construct is longitudinal change in sensor-derived postural control and movement quality, operationalised across four equally ranked primary outcome domains: postural control, weight distribution, movement coordination, and neuromuscular activity. A hierarchy therefore exists between the primary and secondary outcomes, but no further ranking is applied within the four primary outcome domains. All remaining task-performance, daily activity, clinical and patient-reported outcomes are considered secondary. This classification is consistent with the ClinicalTrials.gov record.	We revised the study objectives to explicitly define the primary outcome construct and the four equally ranked primary outcome domains. In Table 1, the **primary outcome domains are now identified** with an asterisk, accompanied by a footnote clarifying that no further hierarchy applies within the primary tier and that all other outcomes are secondary. We also revised the Statistical analysis section to distinguish between analyses of the primary outcome domains and secondary longitudinal analyses. The Sensor-based outcome measures section was additionally revised to describe how the four primary domains are operationalised.
5	**4. I would suggest completing the discussion with some sentences about the expected outcomes and what they could be good for.**<br><br>Moreover, what challenges are to be expected, e.g. it must be a challenge to ask patients to come to the hospital again after 6 months. Is travel reimbursed?	Thank you for this suggestion. We agree that the expected contribution and practical challenges of the study could be described more explicitly. As the manuscript did not previously include a separate Discussion section, **we have added a concise Discussion section.**<br><br>This section describes the expected contribution of FOSTER, including the generation of longitudinal multimodal rehabilitation data, the potential to capture aspects of recovery not fully reflected by clinical scales alone, and the contribution of rehabilitation-related data to the wider TARGET project.<br><br>We also added the main anticipated challenges, including participant burden, variable feasibility of sensor-based tasks across recovery stages, missing data and follow-up after discharge, particularly at 24 weeks. The manuscript now specifies that follow-up after discharge is voluntary, that assessments are scheduled flexibly and aligned with outpatient	A concise Discussion section has been added to the manuscript. This section describes the expected contribution of the FOSTER study and the main anticipated challenges, including participant burden, task feasibility, missing data, voluntary follow-up after discharge, alignment with outpatient rehabilitation appointments where possible, and the absence of travel reimbursement.

		rehabilitation appointments whenever possible, and that travel costs are not reimbursed, which may contribute to loss to follow-up after discharge.	

VERSION 2 - REVIEW

**Reviewer**	**1**
**Name**	**Brunner, Iris**
**Affiliation**	**Aarhus University**
**Date**	**18-Jul-2026**
**COI**	

Looks good now!
