# Supplementary material for: Mapping the longitudinal trajectories of postural control and functional activity after stroke using clinical and sensor-based assessments: protocol of the FOSTER prospective cohort study
Source: BMJ Open. 2026 Aug 17;16(8):e121574. doi: 10.1136/bmjopen-2026-121574 (PMC13504868; doi:10.1136/bmjopen-2026-121574)
Supplement: Supplementary file 2 [file bmjopen-16-8-s001.docx]

**Supplementary materials**

TARGET’s Consortium Author List

Liverpool John Moores University

Sandra Ortega-Martorell^1,2^, ORCID: 0000-0001-9927-3209, EMAIL: s.ortegamartorell@ljmu.ac.uk

Ivan Olier^1,2^, ORCID: 0000-0002-5679-7501, EMAIL: I.A.OlierCaparroso@ljmu.ac.uk

Dharmesh Mistry^1,2^, ORCID: 0000-0001-8300-109X, EMAIL: D.Mistry@ljmu.ac.uk

George Margereson^1,2^, ORCID: 0009-0004-5117-4032, EMAIL: G.H.Margereson@ljmu.ac.uk

Nosa Aikodon^1,2^, ORCID: 0009-0005-9374-7078, EMAIL: arinaiko@ljmu.ac.uk

Robyn Lotto^2^, ORCID: 0000-0002-4305-0513, EMAIL: r.r.lotto@ljmu.ac.uk

Ellen Dawson^2^, ORCID: 0000-0002-5958-267X, EMAIL: e.dawson@ljmu.ac.uk

Costis Maganaris, ORCID: 0000-0002-1786-8047, EMAIL: C.Maganaris@ljmu.ac.uk

Bill Baltzopoulos, ORCID: 0000-0003-2050-9501, EMAIL: V.Baltzopoulos@ljmu.ac.uk

Garry McDowell^2^, ORCID: 0000-0002-2880-5236, EMAIL: G.McDowell@ljmu.ac.uk

**Affiliations:**

^1^ Data Science Research Centre, Liverpool John Moores University, Liverpool, United Kingdom

^2^ Liverpool Centre for Cardiovascular Science at University of Liverpool, Liverpool John Moores University and Liverpool Heart & Chest Hospital, Liverpool, United Kingdom

LUNDS UNIVERSITET:

Mattias Ohlsson^1^, ORCID: 0000-0003-1145-4297, EMAIL: mattias.ohlsson@cec.lu.se

Carsten Peterson^1^, ORCID: 0000-0001-7362-2191, EMAIL: carsten.peterson@cec.lu.se

Anders Bjorkelund^1^, ORCID: 0000-0001-7426-914X, EMAIL: anders.bjorkelund@cec.lu.se

**Affiliations:**

^1^ Computational Science for Health and Environment, Centre for Environmental and Climate Science, Lund University, Lund, Sweden

University of Liverpool:

Gregory Y. H. Lip^1,2,3^, ORCID: 0000-0002-7566-1626, EMAIL: gregory.lip@liverpool.ac.uk

Deirdre A. Lane^1,2,3^, ORCID: 0000-0002-5604-9378, EMAIL: deirdre.lane@liverpool.ac.uk

Yalin Zheng, EMAIL: yzheng@liverpool.ac.uk

Wahbi El-Bouri^1,3^, ORCID: 0000-0002-2732-5927, EMAIL: w.el-bouri@liverpool.ac.uk

Riccardo Proietti, EMAIL: Riccardo.Proietti@liverpool.ac.uk

**Affiliations:**

^1^ Liverpool Centre for Cardiovascular Science at University of Liverpool, Liverpool John Moores University and Liverpool Heart & Chest Hospital, Liverpool, United Kingdom

^2^ Danish Center for Health Services Research, Department of Clinical Medicine, Aalborg University, Aalborg, Denmark

^3^ Department of Cardiovascular and Metabolic Medicine, Institute of Life Course and Medical Sciences, University of Liverpool, Liverpool, United Kingdom

UNIVERSITAT ROVIRA I VIRGILI:

Domènec Savi Puig Valls^1^, ORCID: 0000-0002-0562-4205, EMAIL: doemenc.puig@urv.cat

Hatem Rashwan^1^, ORCID: 0000-0001-5421-1637, EMAIL: hatem.abdellatif@urv.cat

Gaurav Kumar Yadav^1^, ORCID: 0000-0001-7022-290X, EMAIL: gauravkumar.yadav@urv.cat

**Affiliations:**

^1^ Universitat Rovira I Virgili

Hospital Del Mar Research Institute Barcelona:

Esther Duarte Oller, EMAIL: [eduarte@psmar.cat](mailto:eduarte@psmar.cat)

Eva Giralt-Steinhauer, EMAIL: [egiralt@imim.es](mailto:egiralt@imim.es)

Anna Guillen-Sola, EMAIL: aguillen@psmar.cat

Joan Jimenez Balado, EMAIL: joan.balado@gmail.com

Jordi Jimenez Conde, EMAIL: [jjimenez@psmar.cat](mailto:jjimenez@psmar.cat)

**Affiliations:**

IMIM-Hospital del Mar Medical Research Institute, Barcelona, Spain.

Vrije Universiteit Brussel:

Bart Jansen ^3,4^, ORCID: 0000-0001-8042-6834, EMAIL: bart.jansen@vub.be

Lubos Omelina^3^ , ORCID: 0000-0002-2500-5217, EMAIL: lubos.omelina@vub.be

Eva Swinnen^1^, ORCID: [0000-0002-3771-9479](https://orcid.org/0000-0002-3771-9479), EMAIL: eva.swinnen@vub.be

Charlotte Moeyersons^1^, ORCID: 0009-0000-6697-7138, EMAIL: charlotte.moeyersons@vub.be

Sylvie De Raedt^2^, ORCID: 0000-0001-8042-2024, EMAIL: sylvie.deraedt@uzbrussel.be

**Affiliations:**

^1^ Rehabilitation Research Group (RERE), faculty of physical education and physiotherapy, Vrije Universiteit Brussel, Brussels, Belgium

^2^ Department of Neurology, Universitair Ziekenhuis Brussel (UZ Brussel), NEUR Research Group, Vrije Universiteit Brussel (VUB), Brussels, Belgium

^3^ Department of Electronics and Informatics (ETRO), Vrije Universiteit Brussel (VUB), Pleinlaan 2, B-1050 Brussels, Belgium.

^4^ IMEC, Kapeldreef 75, B-3001 Leuven, Belgium.

Revalidatieziekenhuis Inkendaal:

Bernard Dan^1,3^, ORCID: 0000-0002-2051-9876 , EMAIL: bernard.dan@inkendaal.be

Marc Degelaen^1,2^, ORCID: 0000-0001-6651-2496, EMAIL: marc.degelaen@inkendaal.be

Elissa Embrechts^1,2,4^, ORCID: 0000-0002-3039-4579 , EMAIL: elissa.embrechts@inkendaal.be

Britta Hanssen^1,2^, ORCID: 0000-0003-0406-6050, EMAIL: britta.hanssen@inkendaal.be

**Affiliations:**

^1^ Revalidatieziekenhuis Inkendaal, Vlezenbeek, Belgium

^2^ Rehabilitation Research Group (RERE), Faculty of Physical Education and Physiotherapy, Vrije Universiteit Brussel, Brussels, Belgium

^3^ Université libre de Bruxelles (ULB), Faculty of Psychology, Educational Sciences and Speech and Language therapy (CP122), 50 Avenue F. Roosevelt, 1050 Brussels, Belgium

^4^ Movement Antwerp (MOVANT), Department of Rehabilitation Sciences and Physiotherapy, Faculty of Medicine and Health Sciences, Wilrijk, Belgium.

Institut National Polytechnique de Toulouse:

Sylvie Chambon

**Affiliations:**

Institut National Polytechnique de Toulouse

Siemens SRL:

Lucian Mihai Itu^1^, ORCID: 0000-0002-2205-497X, EMAIL: lucian.itu@siemens.com

**Affiliations:**

^1^ Siemens SRL, Advanta, Brasov, Romania

Universitatea de Medicina, Farmacie, Stiinte Si Tehnologie Din Targu Mures:

Theodora Benedek^1^ , ORCID: 0000-0001-8326-1537, Email: [theodora.benedek@gmail.com](mailto:theodora.benedek@gmail.com)

Imre Benedek^1^, ORCID: 0000-0003-0051-4047, Email: [imrebenedek@yahoo.com](mailto:imrebenedek@yahoo.com)

Nora Rat^1^, ORCID: 0000-0001-6975-5987, Email: [ratnora@gmail.com](mailto:ratnora@gmail.com)

Emanuel Blindu^1^, ORCID: 0009-0000-1229-3697, Email: [emi.blindu@yahoo.com](mailto:emi.blindu@yahoo.com)

Ioana Rodean^1^, ORCID: 0000-0001-7234-0088, Email: [ioana_patricia91@yahoo.com](mailto:ioana_patricia91@yahoo.com)

Bogdan Halatiu^1^, ORCID: 0000-0001-6793-9248, Email: [bhalatiu@yahoo.com](mailto:bhalatiu@yahoo.com)

Renata Gerculy^1^, ORCID: 0000-0003-1105-930X, Email: [gerculy_renata@yahoo.com](mailto:gerculy_renata@yahoo.com)

Aurelian Rosca^1^, ORCID: 0009-0000-5275-7265, Email: [rosca_aurelian@yahoo.com](mailto:rosca_aurelian@yahoo.com)

Theofana Mihaila^1^, ORCID: 0009-0001-9196-4491, Email: [theofana_m@yahoo.com](mailto:theofana_m@yahoo.com)

**Affiliations:**

^1^ George Emil Palade University of Medicine, Pharmacy, Science and Technology of Târgu Mureș

Radboud University Medical Center:

Anil Tuladhar, EMAIL: Anil.Tuladhar@radboudumc.nl

Tim ten Cate, EMAIL: Tim.tenCate@radboudumc.nl

**Affiliations:**

Radboud University Medical Centre, Donders Institute for Brain, Cognition and Behavior, Nijmegen, the Netherlands

Liverpool Heart and Chest Hospital:

Timothy Fairbairn^1,2^, ORCID: 0000-0003-1491-6231, EMAIL: timothy.fairbairn@lhch.nhs.uk

Laurence Tidbury^1,2^, ORCID: 0000-0002-7748-8791, EMAIL: laurence.tidbury@lhch.nhs.uk

**Affiliations:**

^1^ Department of Cardiology, Liverpool Heart and Chest Hospital, Liverpool, United Kingdom

^2^ Liverpool Centre for Cardiovascular Science at University of Liverpool, Liverpool John Moores University and Liverpool Heart & Chest Hospital, Liverpool, United Kingdom

Arrhythmia Alliance:

Trudie Lobban^1,2,3^, ORCID: 0000-0002-1655-0387, EMAIL: trudie@heartrhythmalliance.org

**Affiliations:**

^1^Arrhythmia Alliance

^2^AF Association

^3^STARS (Syncope Trust And Reflex anoxic Seizures), 55 Dean Lane, Winchester, Hampshire SO22 5JR, UK

Liverpool University Hospitals NHS Foundation Trust:

Ingeborg Welters^1^, EMAIL: I.Welters@liverpool.ac.uk

Brian Johnston^1^, EMAIL: Brian.Johnston@liverpool.ac.uk

Hani Essa^1^, EMAIL: hani.essa@liverpoolft.nhs.uk

**Affiliations:**

^1^ Liverpool Centre for Cardiovascular Science at University of Liverpool, Liverpool John Moores University and Liverpool Heart & Chest Hospital, Liverpool, United Kingdom

Technische Universitaet Wien:

Katja Hose^1^, ORCID: 0000-0001-7025-8099, EMAIL: katja.hose@tuwien.ac.at

Renata G. Raidou^1^, ORCID: 0000-0003-2468-0664, EMAIL: rraidou@cg.tuwien.ac.at

Milos Jovanovik^1^, ORCID: 0000-0001-7360-8015, EMAIL: milos.jovanovik@tuwien.ac.at

Tomer Sagi^1,2^, ORCID: 0000-0002-8916-0128, EMAIL: tomer.sagi@tuwien.ac.at

**Affiliations:**

^1^ TU Wien, Vienna, Austria

^2^ Aalborg University, Denmark

Siemens Healthineers AG:

Oliver Frings^1^, ORCID: 0000-0001-7087-8060, EMAIL: oliver.frings@siemens-healthineers.com

Eric Lluch^1^, ORCID: 0000-0003-0732-4385 , EMAIL: eric.lluch@siemens-healthineers.com

**Affiliations:**

^1^ Siemens Healthineers AG, Digital Technology and Innovation, Erlangen, Germany

Moverim Consulting SRL:

Laura Vivani, EMAIL: vivani@moverim.eu

Veronica Christofidis, EMAIL: [christofidis@moverim.eu](mailto:christofidis@moverim.eu)

Silvia Anastasia, EMAIL: anastasia@moverim.eu

**Affiliations:**

MOVERIM CONSULTING SRL

Center for Research and Technology Hellas:

Dimitrios Tsaopoulos, ORCID: 0000-0001-7355-0511, EMAIL: d.tsaopoulos@certh.gr

Spiros Nikolopoulos, ORCID: 0000-0002-1367-5133, EMAIL: nikolopo@iti.gr

Konstantinos Risvas, ORCID: 0000-0001-9177-9301

Fotis Kalaganis, ORCID: 0000-0002-5474-9098, EMAIL: fkalaganis@iti.gr

Dimitrios-Sokratis Komaris, EMAIL: s.komaris@iti.gr

**Affiliations:**

CENTER FOR RESEARCH AND TECHNOLOGY HELLAS
